# Optimizing the Correction of Depressive Disorders in Patients with Primary Hypothyroidism

**DOI:** 10.1192/j.eurpsy.2023.858

**Published:** 2023-07-19

**Authors:** O. M. Pityk

**Affiliations:** Psychiatry, Narcology and Medical Psychology, Ivano-Frankivsk National Medical University, Ivano-Frankivsk, Ukraine

## Abstract

**Introduction:**

Population recent studies have shown that most patients with endocrine pathology suffer from at least one of the three DCPR syndromes: irritable mood, demoralization (despair), persistent somatization. The thyroid gland is a unique organ among the glands of internal secretion, in the pathology of which non-psychotic mental disorders are extremely common. Therefore, the use of a complex, integrative, systemic approach in the examination of patients with thyroid pathology should be the basis of planning the strategy and tactics of the treatment program for such patients.

**Objectives:**

We examined 132 patients with primary hypothyroidism.

**Methods:**

We used psychopathological method and an adapted methodology for assessing typologies of psychological defence. It was the method of Robert Plutchik adapted by L.I.Wasserman, O.F.Eryshev, E.B.Klubova for assessment of the next mechanisms of defense: negation, projection, regression, displacement, repression, intellectualization, reactive formation, compensation.

**Results:**

In 108 patients, who made up 81.12% of the total number of investigated, various forms of non-psychotic mental disorders were detected, among which 23 patients (12.04%) had an anxiety-depressive syndrome, and astheno-depressive syndrome (32.41%).

It was established that excessive compensation, projection, reactive formation formed a tendency to increased self-control, to analysis and introspection, self-justification, isolation, which in general influenced the structure of the astheno-depressive syndrome. Insufficient reactive formation, displacement, and excessive intellectualization in a complex contributed to the formation of subjective feelings of anxiety and fear in patients, led to the avoidance of problematic situations, unnatural slowness with behavioral manifestations of anxiety, therefore influenced the structuring of anxiety and depressive disorders at the same time. Thus, significant connections have been established between the intrapsychic level of functioning and the formation of astheno-depressive and anxiety-depressive disorders, which should be used in the planning of psychotherapeutic and psychocorrective measures.

**Conclusions:**

This approach ensures that endocrinologists and general practitioners master the simplest skills for providing psychocorrective care to patients with depressive disorders. It includes the application of elements of rational psychotherapy, in order to form a sense of control over their own condition and the ability to master negative influences, which contributes to stabilization their general condition and improvement of the quality of life. So medical care to such patients should focus on early diagnosis and correction of nonpsychotic mental disorders. Both medications and psychological influences should be used in the treatment of such patients.

**Disclosure of Interest:**

None Declared

